# Towards an Understanding of Microglia and Border-Associated Macrophages

**DOI:** 10.3390/biology12081091

**Published:** 2023-08-05

**Authors:** Takumi Taketomi, Fuminori Tsuruta

**Affiliations:** 1 PhD Program in Human Biology, School of Integrative and Global Majors, University of Tsukuba, Tsukuba 305-8577, Japan; s2130510@u.tsukuba.ac.jp; 2 Master’s and Doctoral Programs in Biology, Faculty of Life and Environmental Sciences, University of Tsukuba, Tsukuba 305-8577, Japan; 3 PhD Program in Humanics, School of Integrative and Global Majors, University of Tsukuba, Tsukuba 305-8577, Japan; 4 Master’s and Doctoral Program in Neuroscience, Graduate School of Comprehensive Human Sciences, University of Tsukuba, Tsukuba 305-8577, Japan

**Keywords:** microglia, border-associated macrophages, neurodegeneration, neuroinflammation, aging, development, homeostasis, brain parenchyma, meninges, neurovascular space, choroid plexus

## Abstract

**Simple Summary:**

In the central nervous system, immune cells can be broadly categorized as parenchymal microglia or non-parenchymal border-associated macrophages. It has become increasingly evident in recent years that these cells have unique functions beyond their roles in inflammatory responses under pathological conditions. Investigating these cells can provide valuable insights into how microglia and macrophages regulate brain function throughout life, including developmental, homeostatic, aging, and disease states. Here, we provide a comprehensive overview of the current understanding of these cells in the brain.

**Abstract:**

The central nervous system (CNS) plays a crucial role in regulating bodily functions by sensing and integrating environmental cues and maintaining proper physiological conditions. Recent research has revealed that CNS functions are closely coordinated with the immune system. As even minor disturbances of the immune system in the CNS can lead to various dysfunctions, diseases, or even death, it is highly specialized and segregated from that in peripheral regions. Microglia in the parenchyma and macrophages at the interface between the CNS and peripheral regions are essential immune cells in the CNS that monitor environmental changes. Recent omics analyses have revealed that these cells exhibit highly heterogeneous populations. In this review, we summarize the functions and diversity of microglia in the brain parenchyma and those of macrophages in the border regions, such as the meninges, perivascular spaces, and choroid plexus.

## 1. Introduction

Microglia, the resident immune cells in the brain parenchyma, play a pivotal role in establishing optimal brain environments by regulating apoptotic cells clearance [1], neurogenesis [2], synaptogenesis [3], synapse pruning [4,5], neuronal differentiation [6,7], neuronal survival [8], synaptic surveillance [9], extracellular matrix maintenance [10,11], and vascular functions [12,13]. Microglia originate from early erythromyeloid progenitors (EMPs) in the yolk sac and migrate to the brain primordium through circulation [14]. After infiltration, these cells differentiate into various types of microglia. Recent advances in single-cell RNA sequencing (scRNA-seq) technology have made microglial temporal and spatial heterogeneities hot topics in this field. The gene expression signature in homeostatic microglia [15] differs from those in developmental [16,17,18], aging [19,20], and disease microglia, including neurodegenerative disorders [21,22,23,24,25] and brain tumors [26]. In addition to microglia, omics analyses have unveiled the existence of various macrophages in the border region, known as border-associated macrophages (BAMs) or CNS-associated macrophages (CAMs) (hereafter referred to as BAMs) [27,28,29,30]. BAMs reside in the non-parenchymal CNS interface, such as the meninges, perivascular region (Virchow-Robin space), and choroid plexus. BAMs share some gene expression signatures with microglia in the brain parenchyma but also have distinct and characteristic gene expression profiles. Recent findings suggest that the differences in transcriptome profiles between microglia and BAMs are due to differences in their origin or niche [31].

**Figure 1:**
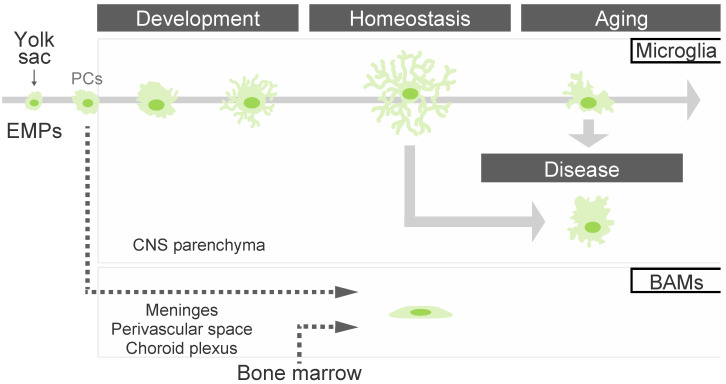
Changes in the state of microglia and macrophages in the brain. During embryogenesis, cells derived from the yolk sac differentiate into microglia in parenchyma and border-associated macrophages (BAMs) in CNS border regions, such as the meninges, perivascular regions, and choroid plexus. The functions and characteristics of microglia and BAMs are diverse and contextually dependent. It has been reported that microglia undergo functional changes concomitant with morphological changes. However, there is insufficient information regarding the ontogeny of BAMs. PCs: microglial and BAM precursor cells (MG/BAM PCs).

It is thought that there are two transcriptionally distinct populations within the EMPs population, which serves as a common progenitor for both microglia and BAMs. These populations are distinguished by the expression of BAMs-specific marker CD206 [32], implying that the cell fates of microglia and BAMs depend on the CD206 expression and timing of EMP differentiation. Recent fate mapping analysis revealed that CD206^+^ cells within the EMP population could differentiate into both microglia and BAMs [33]. Additionally, CD206^+^ macrophages infiltrating from the ventricles can convert into microglia within the brain parenchyma [34]. These findings suggest that the fate determination of microglia and BAMs is niche-dependent, and thus these diverse lineages can be assumed to contribute to the generation of microglial diversity. Here, we outline the diverse functions of microglia in the brain parenchyma and also provide an overview of BAMs function in various brain regions, such as the meninges, perivasculature space, and choroid plexus (Figure 1). During embryogenesis, cells derived from the yolk sac differentiate into microglia in parenchyma and BAMs in CNS interfaces, such as the meninges, perivascular regions, and choroid plexus. The functions and characteristics of microglia and BAMs are diverse and contextually dependent.

## 2. The State of Microglia in the Brain Parenchyma

The mammalian brain can be divided into two parts: parenchyma and non-parenchyma. The brain parenchyma, which plays a central role in brain function, comprises not only neurons but also glia, including microglia, astrocytes, and oligodendrocytes. Recently, it has been shown that microglia in the brain parenchyma undergo dynamic changes depending on developmental, homeostatic, aging, and disease states, thereby influencing higher brain functions. Here, we introduce the new functions and roles of microglia that have been identified in recent years.

### 2.1. Microglial Differentiation and Maturation

During embryonic development, microglial progenitor cells migrate into the brain primordium via the bloodstream, where they undergo differentiation and maturation to become homeostatic microglia. These microglia exhibit dynamic behavior throughout this process by responding to diverse stimuli from their surrounding environment. The EMPs derived from the yolk sac differentiate into microglial and BAM precursor cells (hereafter abbreviated as MG/BAM PCs) (Figure 1) in Myb-independent machinery, contrary to bone marrow–derived macrophages [27,35]. MG/BAM PCs subsequently differentiate into reactive microglia via crucial factors such as interferon regulatory factor 8 (IRF8) [36]. Previous studies have identified various signaling pathways involved in microglial differentiation and survival. Among them, transforming growth factor β (TGFβ) signaling has been suggested as crucial for microglial, maturation and maintenance for homeostatic microglia, but not BAM [32]. Indeed, the loss of the *Tgfbr2* gene in hematopoietic cells, which give rise to inhibition of TGFβ signaling in MG/BAM PCs, attenuates the gene expressions involved in regulating microglial signature but has little effect on BAMs. Similarly, impairment of the *Smad4* gene, a downstream factor of TGFβ signaling, during early development in MG/BAM PCs suppresses microglial differentiation and maturation, despite the minimal effect on BAM differentiation [37]. As microglial TGFβ signaling also maintains the homeostatic state in vitro and in vivo [38,39], this signaling plays a pivotal role in microglial maturation and homeostasis. Importantly, several highly expressed genes in homeostatic microglia such as *Csf1r* and *Cx3cr1* are expressed during the fetal stage in the human embryonic brain, contributing to the acquisition of homeostatic microglial characteristics [18]. As there are differences in the maturation process of microglia between primates and rodents, future research from an evolutionary perspective is necessary and should prove to be an exciting topic.

So, what types of microglia exist after birth? They exhibit a highly diverse cellular state during the postnatal period. Recent omics analyses have identified the presence of multiple states, even in physiological conditions during development. For instance, a state known as proliferative region–associated microglia (PAM) emerges near white matter around P7 but not embryonic (E14.5) and adult (P60) stages, and is involved in the phagocytosis of oligodendrocytes [16]. Deep scRNA-seq analyses have revealed that the expression of disease-associated microglia (DAM) marker genes, including *Spp1*, *Gpnmb*, and *Clec7a*, was increased in PAMs. In contrast to DAM, the regulation of these genes occurs in a triggering receptor expressed in a myeloid cells 2 (TREM2)-apolipoprotein E (ApoE) pathway–independent manner in PAM. Moreover, another subpopulation, referred to as axon tract–associated microglia (ATM), forms a new cluster near P4/P5 and exhibits strong expression of *Spp1*, *Igf1*, and *Gpnmb* [17]. These microglia are also observed near subcortical axons. Given the abundance of ATM in axons, it is plausible that ATM play roles in both myelination and the clearance of myelin debris. However, the observation that numerous ATM are lost prior to myelination suggests another possibility for regulating axon function.

### 2.2. The Microglial State in Aging and Diseases

Microglial diversity has been extensively researched not only during the postnatal period but also in the context of diseases and aging. In particular, omics analyses have demonstrated that distinct subpopulations of microglia appear during the normal aging process. Other states have also been found to emerge in neurodegenerative diseases such as Alzheimer’s disease (AD), Parkinson’s disease (PD), and amyotrophic lateral sclerosis (ALS), as well as autoimmune diseases like multiple sclerosis (MS). For instance, the DAM subpopulation of microglia has been identified in mouse models of AD [21]. DAM is a phagocytic state of microglia that emerges during pathological conditions. There are two distinct stages. The first stage undergoes a transition from a homeostatic state in a TREM2-independent manner and subsequently progresses to the second stage through a TREM2-dependent mechanism. It has been shown that genes such as *Cx3Cr1*, *P2ry12*, and *Tmem119* are highly expressed in homeostatic microglia. However, the amount of these genes decreases in the first stage, while the expression of genes such as *Ctsb*, *Ctsd*, and *Apoe* increases. In the second stage, the expression of genes such as *Trem2*, *Axl*, *Ctsl7*, and *Clec7a* is further upregulated. These alterations in gene expression profiles lead to the production of microglia that exhibit characteristic features of the underlying pathology. Additionally, they share various gene expression profiles with not only PAM and ATM, but also the white matter–associated microglia (WAM) responsible for the clearance of degenerated myelin in aging brains [16,17,19]. In addition to DAM, other types of microglia, such as microglial neurodegenerative phenotype (MGnD) [22], activated-response microglia (ARM), and interferon response microglia (IRM) [40], have been reported in various subpopulations, including those with AD, MS, and ALS [18,23,25]. scRNA-seq analyses have further expanded the diversity of microglial states to include glioma-associated microglia (GAM) in glioblastoma [26]. *Itgal/Cd11a* has been identified as a marker for GAM. Ablation of the *Itgal/Cd11a* gene attenuates the aberrant proliferation of glioblastoma, suggesting that CD11a is a novel therapeutic target for this tumor. Taken together, these findings indicate that microglial states in the brain parenchyma are not homogenous entities but rather comprise a complex mixture of diverse subpopulations that collectively form a heterogeneous population (Table 1).

### 2.3. Microglial Functions in the Homeostatic State

Previous studies have suggested that microglia exhibit high dynamics during both development and aging. On the other hand, it was previously thought that mature homeostatic microglia are quiescent under normal conditions and are only activated when foreign antigens invade the brain. However, observations using two-photon microscopy have shown that microglia dynamically change their morphology by repeatedly extending and retracting their processes even under normal physiological conditions [41].

Microglia dynamically monitor synaptic connectivity and foreign substances, regulating brain homeostasis. Interestingly, even homeostatic microglia, which do not exhibit significant differences in gene expression signatures, acquire unique niche-dependent functions under certain conditions. The scRNA-seq technique has yielded significant insights into various aspects of microglial biology, including their activation states. However, other research approaches, such as direct observation of mouse brain and cell-based assays, have also contributed to our understanding of homeostatic microglia and their functions.

Recent studies have revealed that microglia play a crucial role not only in regulating synaptic connectivity but also in maintaining brain vessels. Indeed, almost half the microglia are attached to brain wall capillaries [42]. Specifically, microglia have a role in regulating blood flow in the vessels and preserving the integrity of the blood–brain barrier (BBB). For example, the function of microglia around blood vessels in the brain parenchyma is regulated by P2Y purinoceptor 12 (P2RY12), which accepts extracellular ATP [13]. In particular, the depletion of microglia with PLX3397, a specific inhibitor of colony-stimulating factor receptor (CSFR), causes increased cerebral blood flow, microvascular dilation, and impaired vasodilation [13]. At a steady state, microglia are in contact with the BBB and maintain its integrity. However, when an inflammatory response is triggered, microglia phagocytose the endfeet of astrocytes, thereby reducing the function of the BBB [12]. Recently, it was reported that the interaction between microglia and perivascular pericytes regulates microglial survival [42], suggesting that microglia may be closely involved in forming and maintaining cerebrovascular function.

Another function of microglia that has become clear in recent years is the regulation of oligodendrocytes. Oligodendrocytes wrap around axons of neurons after birth and form myelin. During a specific timing of postnatal development around P7, a subpopulation of microglia known as PAM reside in the white matter of the brain and remove unnecessary myelin, subsequently contributing to normal myelin formation [16]. In addition, the depletion of microglia leads to a downregulation of TGFβ levels and the degeneration of myelin in the white matter, resulting in the onset of cognitive impairment [43]. Furthermore, the loss of oligodendrocyte-specific TGFβR signaling causes abnormal myelin and axon formation [43], suggesting that TGFβ signaling within myelin is required to maintain normal myelin formation. Because remyelination following neuroinflammation is also reliant on microglia [44], it is reasonable to consider that the regulation of oligodendrocytes by microglia is crucial for maintaining brain homeostasis, and that the TGFβ signaling pathway may serve as an important mechanism in both processes.

Recent research has shown that microglia play a significant role in regulating higher brain functions by clearing the extracellular matrix. For instance, adult hippocampal neurons expressing interleukin 33 (IL33) activate IL33 receptors on microglia, leading to enhanced phagocytosis of the surrounding extracellular matrix and subsequent synaptic remodeling [10]. Additionally, microglia are shown to degrade extracellular matrix structures, such as perineuronal nets, in the dorsal horn of the spinal cord, thereby regulating pain sensation [11]. After peripheral nerve injury, microglia-induced perineuronal net degradation activates projection neurons that exacerbate sensitivity to pain. Thus, microglia contribute to pain sensation by activating nociceptive circuits in the spinal cord through extracellular matrix degradation [45]. However, pain-remitting CD11c^+^ microglia have also been found in the spinal cord. These microglia express insulin-like growth factor-1 (IGF1) and contribute to pain attenuation. A close relationship between the pain response and the inflammatory response has been suggested, and it can be speculated that the induction of an inflammatory response in the microenvironment may enhance the pain response. Intriguingly, microglia produce various extracellular matrix components, including secreted protein acidic and rich in cysteine (SPARC), which has been suggested as a factor dominantly expressed in microglia [29,37,46]. SPARC not only functions as an extracellular matrix but also induces inflammatory responses by binding to Toll-like receptor 4 (TLR4) [47]. Given that the formation of an appropriate extracellular matrix is essential for regulating inflammatory responses in the microenvironment, it could be possible that a microglia-regulated extracellular matrix formation may influence controlling nociceptive circuits.

## 3. The Roles of Border-Associated Macrophages

In addition to brain parenchymal microglia, there are also BAMs residing in the non-parenchyma regions, such as the meninges, perivascular areas, and choroid plexus. BAMs have similar properties to microglia but differ in their detailed signature. In this section, we outline the non-parenchymal region briefly and introduce the lineage and novel roles of BAMs that were reported in the past few years.

### 3.1. Meningeal Macrophages

**Figure 2:**
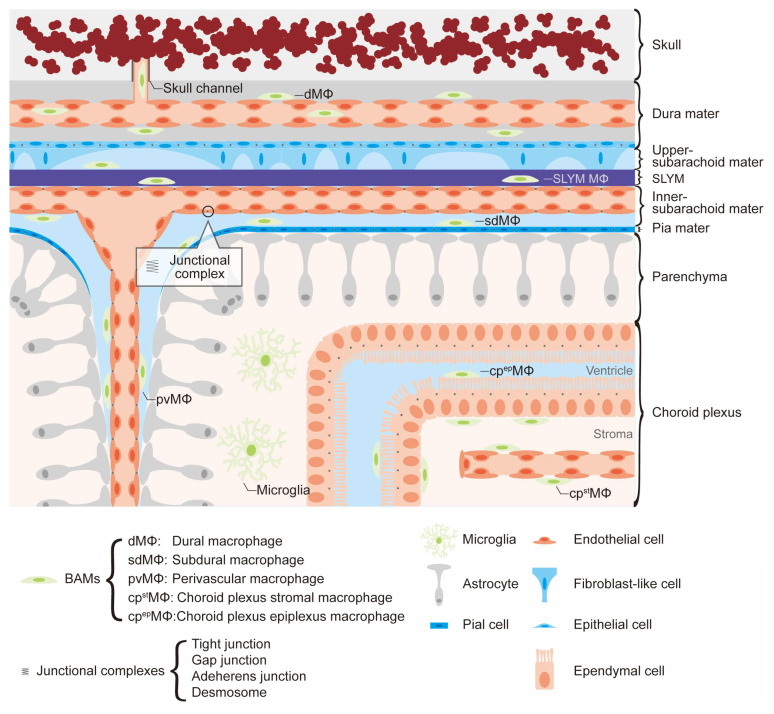
BAMs population in CNS border regions. CNS border regions are highly specialized anatomical structures. BAMs have heterogeneous populations to adapt to each CNS border region, such as the perivascular regions, choroid plexus, and meninges (dura mater, arachnoid mater, pia mater). The dura mater and choroid plexus have fenestrated vessels, allowing replacement by bone marrow–derived macrophages. Similarly, SLYM is connected to the venous sinus wall, allowing infiltration of bone marrow–derived macrophages. Skull channels are the direct vascular channels that connect the skull bone marrow and dura mater, allowing immune cell migration. Ventricle, subarachnoid space, and perivascular regions are filled with cerebrospinal fluid (CSF), dMΦ, dural macrophage, SLYM, subarachnoid lymphatic-like membrane, sdMΦ, subdural macrophage, pvMΦ, perivascular macrophage, cp^st^MΦ, choroid plexus stromal macrophage, and cp^ep^MΦ, choroid plexus epiplexus macrophage.

Meningeal macrophages are the resident macrophages in the meninges, consisting of the dura mater, arachnoid mater, and pia mater. Transcriptome analysis has implied that multiple subsets of meningeal macrophages exist in the superficial and deep layers of the meninges. These observations demonstrate that meningeal macrophages could be subdivided due to their unique anatomical compartments (Figure 2) [29].

The dura mater is the thick outermost layer, closely associated with the skull. The dura mater has fenestrated blood vessels lacking tight junctions, which allows exposure to solutes from the circulation. The resident macrophages in the dura mater, called dural macrophages, initially originate from EMPs but are replaced by monocyte-derived macrophages in an age-dependent manner [29]. Interestingly, dural monocyte–derived macrophages mainly originate from skull bone marrow through the skull channels, which are composed of vascular channels connecting the skull bone marrow to the dura mater [48].

The arachnoid mater is an intermediate layer consisting of two parts: the avascular connective tissue beneath the dura mater and the arachnoid trabeculae extending towards the pia mater. The avascular connective tissue consists of epithelial cells, and the trabeculae consist of fibroblast-like cells and sheet-like collagen, forming the subarachnoid space. Subdural meningeal macrophages, also known as leptomeningeal macrophages, reside in the subarachnoid space, which is composed of the arachnoid mater and pia mater. It is noteworthy that, unlike dural macrophages, these subdural meningeal macrophages do not transform from monocytes under homeostatic conditions [29]. The subarachnoid space also contains cerebrospinal fluid (CSF) and blood vessels that supply the brain parenchyma, resulting in the direct connection with the perivascular areas, also known as Virchow–Robin spaces [49].

The pia mater is the innermost and thinnest layer of the meninges, closely adhering to the CNS parenchyma. This delicate and continuous layer is composed of fused cells that are interconnected by gap junctions and desmosomes, which limit the movement of macromolecules between the parenchyma and the CSF, but do not affect the small molecules and solutes [50,51]. The pia mater is penetrated by subarachnoid vessels, allowing macrophages to migrate from the meninges to the perivascular region.

Recently, a fourth membrane in the meninges, known as the subarachnoid lymphatic–like membrane (SLYM), was identified [52]. The SLYM is situated between the arachnoid and the pia mater. The gene expression signature of SLYM cells exhibits characteristics similar to those of the lymphatic marker, *Pdpn*, but not *Lyve1*. Although the precise biological significance of the SLYM is still being investigated, it may play a role in facilitating the transfer of small molecules between the CSF and venous blood. Notably, myeloid cells were identified within the SLYM, and their abundance increases in both lipopolysaccharide (LPS)-treated and aged mice. For instance, the SLYM was found to contain CD206^+^ and Lyve1^+^ macrophages with dendritic cells. The presence of resident CD206^+^ and Lyve1^+^ macrophages and dendritic cells within the SLYM suggests the existence of a novel subset of BAMs composed of these myeloid cells.

Meningeal macrophages are believed to play a role in the aging process. As previously stated, dural macrophages are replaced by bone marrow–derived (BM) macrophages as part of an age-dependent process [29]. Additionally, major histocompatibility complex class II^-^ (MHC-II^-^) dural macrophages are prevalent in neonates, while MHC-II^+^ dural macrophages increase over time [29]. It has been predicted that replaced BM macrophages might lose their drainage function since drainage efficiency decreases with age. Interestingly, MHC-II^+^ cells, which are relatively enriched by blood-derived monocytes, have demonstrated a greater ability to uptake substances. However, since drainage also involves the decomposition and emission of substances into the circulatory system, further research is necessary to fully understand the mechanisms involved.

Recent studies have shown that meningeal macrophages play a crucial role in protecting the CNS from viral infections [53]. Depletion of meningeal macrophages, either genetically or pharmacologically, leads to increased susceptibility to fatal meningitis induced by lymphocytic choriomeningitis virus, highlighting their importance in antiviral defense. Notably, the antiviral function of meningeal macrophages requires interferon responses rather than MHC-II-mediated antigen presentation. However, the specific populations of meningeal macrophages, such as those that are MHC-II^+^ or MHC-II^-^, subdural or dural, critical for antiviral protection, have yet to be determined.

### 3.2. Perivascular Macrophages

Perivascular macrophages reside in the perivascular (Virchow-Robin) space between endothelial or mural cells and astrocyte end-feet. The perivascular space does not exist when the brain vessels form but emerges after birth. Indeed, transmission electron microscopy observation revealed that perivascular spaces are apparent from P3 to P10 [33]. Also, several studies using reporter mice revealed that perivascular macrophages are derived from subdural meningeal macrophages, which initially originate from yolk sac EMP, occurring around P10 [27,32,33,53]. Perivascular macrophages in the brain have the ability to detect signals of inflammation. The binary Cre system revealed that the transcriptome of BAMs, which are believed to include perivascular macrophages, exhibits an earlier response to the LPS challenge than microglia [54]. It has been reported that perivascular macrophages play a significant role in the pathogenesis of cerebral malaria by regulating invasion spaces for CD8^+^ T cells, ultimately leading to encephalitis. Indeed, in vivo imaging analysis has suggested that perivascular macrophages interact with CD8^+^ T cells, potentially serving as sites for antigen presentation [55]. Similarly, a recent study has suggested that BAMs MHC-II are required for T cells infiltration in an α-synuclein model of PD, indicating that BAMs MHC-II-dependent antigen presentation is a clue for T cell infiltration [56]. However, several studies suggest that microglia and dendritic cells permit T cell infiltration into the brain parenchyma of an AD mouse model with tau mutations and an experimental autoimmune encephalomyelitis model, respectively [57,58]. Although context-dependent cell type changes and the precise mechanism will be an intriguing field for investigation, the perivascular macrophages may represent an initial mediator of the pathogen challenge.

The BBB is a highly selective semipermeable border preventing solutes from circulating blood to the brain parenchyma. Brain perivascular macrophages are thought to regulate the BBB through their contribution to blood permeability. Notably, depletion of perivascular macrophages in the inner ear was demonstrated to result in an increased entry of solutes from the blood, emphasizing their importance in maintaining barrier function [59,60]. Interestingly, the restoration of blood permeability was observed following the repopulation of macrophages treated with interleukin-4, rather than LPS [60]. However, studies on hypertension model mice show that perivascular macrophages can increase blood permeability [61]. These reports suggest that perivascular macrophages have reversible functions on the BBB in a context-dependent manner.

The lymphatic pathway subserves the CSF flow, which is important for fluid-clearance of wastes in the brain [62]. The CSF flow is known to be promoted by aquaporin 4 (AQP4) water channels on the perivascular astrocyte end-feet [63]. A recent study showed that the depletion of perivascular macrophages by clodronate reduces the area of the perivascular region and CSF flow, suggesting that the perivascular macrophages contribute to the perivascular matrix formation and regulate CSF flow [64]. In addition, the decreasing number of CD206^+^ and Lyve1^+^ perivascular macrophages correlates with the decreasing CSF flow efficiency in aging mice, suggesting that the specific type of perivascular macrophages contributes to the CSF flow. Since the Lyve1^+^ macrophages originated from yolk sac–derived EMP, the function of CSF flow regulations may depend on the origin of perivascular macrophages [65]. Perivascular macrophages are also reported to have a function of removing perivascular waste, such as amyloid beta, which is a key molecule for AD [64]. This effect was confirmed by the BAM deletion agent, clodronate, and possible activation agent, chitin [64,66]. Collectively, perivascular macrophages contribute to CNS homeostasis by removing waste on the brain interface.

### 3.3. Choroid Plexus Macrophages

Choroid plexus macrophages are the resident macrophages in and on the choroid plexus. The choroid plexus is a complex network of capillaries lined by specialized epithelial and has a role in CSF production and the blood–CSF barrier (BCSFB) [67,68] (Figure 2). The choroid plexus macrophages are reported to initially originate from EMP and to be replaced with bone marrow–derived macrophages in an age-dependent manner [27]. In addition, it is reported that choroid plexus macrophages are subdivided into stromal and epiplexus macrophages, also known as Kolmer cells [27,69]. Stromal macrophages are largely immobile of the cell body and play a role in vascular surveillance of the choroid plexus with their processes [69]. Epiplexus macrophages are mobile on the apical surface of the choroid plexus. The integrity of BCSFB is essential for brain homeostasis. Recently, in vivo imaging analysis revealed that the choroid plexus macrophages move to the injured vessel region in the choroid plexus [69]. In addition, the peripheral LPS challenge induces the increase of stromal macrophages covering the region [69]. The choroid plexus produces CSF and relates with the hydrocephalus. A recent study using CellChat analysis reported that choroid plexus macrophages induce pathogenic CSF production in a hydrocephalus mice model [70,71]. Thus, choroid plexus macrophages play a key role in CSF homeostasis against inflammation cues.

## 4. Conclusions

Recent advances in experimental methods, such as scRNA-seq, lineage-tracing tools, and in vivo imaging techniques, have enabled us to obtain insights into the origin, turnover, diversity, and functions of microglia and BAMs. Studying the niche signals will provide a better understanding of the maturation of microglia and BAMs. In particular, non-cell autonomous interactions could be critical factors for differentiating macrophages in each niche. Microglia and BAMs are crucial cells that monitor CNS homeostasis. The state alteration of microglia and BAMs during aging is another fascinating research area. Elucidating the state controls of microglia and BAM will provide a better understanding of the pathogenesis mechanism of neurodegenerative disease. Analyzing these critical questions will provide a novel therapeutic approach targeting microglia and macrophages in the CNS.

## Figures and Tables

**Table 1:** Parenchymal microglial state characterized by scRNA-seq analysis.

Name/Description	Abbr.	Subjects	State	Analyzed Stages	Characteristics of Typical Gene Signature	Signatures and Functions	Ref.
Proliferative-region-associated microglia	PAM	Mice	Development	Early postnatal stage (P7) Embryonic stage (E14.5) Adult (P60)	*Gpnmb, Spp1, Clec7a, Itgax, Lilrb4*	PAM appear in the developing corpus callosum and white matter around P7. PAM share a gene signature with DAM. PAM engulf excess oligodendrocytes. PAM appearance does not depend on a TREM2-APOE axis.	[16]
Axon tract-associated microglia Injury-responsive microglia	ATM IRM	Mice	Development Disease	Embryonic stage (E14.5) Early postnatal stage (P4, P5) Adult (P30, P100 ) Aging (P540) Injury (lysolecithine stimulation)	*ARM; Spp1, Igf1, Gpnmb, Lgals1, Lgals3,Lamp1, Cd68, Fabp5* *IRM; Birc5, Cxcl10, Ccl4, Apoe, Ifi27l2a, Ifi204*	ATM appear in axon tracts of the corpus callosum and cerebellum around P4/P5 ATM disappear before myelination occurs. IRM appear after injection of lysolecithin. Gene expression signature in IRM is partially common to DAM and ATM.	[17]
Human fetal brain microglia	_	Human	Development	Embryonic stage (GW 9–18)	*Csf1r, Cx3cr1, P2ry12, P2ry13, Tmem119, Axl, Apoe, Cd68, Mrpl23, Parp4, Mtx1, Hba/Hbg, Zp3, Nampt*	The developmental human microglia are similar to DAM/MGnD in mice. Microglia transform to a more mature, immune-responsive phenotype during embryonic periods.	[18]
White matter-associated microglia	WAM	Mice	Aging	2, 6, 12, 18, 20 and 24 months old	*ApoE, Cst7, Bm2, Lyz2, Cd63, Clec7a, Ctsb, Ctss, Ctsz, H2-D1, H2-K1*	WAM phagocytose damaged myelin in aging white matter. WAM share a gene signature with DAM. WAM appearance depends on a TREM2-dependent but ApoE-independent manner.	[19]
Lipid-droplet accumulating microglia	LDAM	Human Mice	Aging	Human; <35 years old, >60 years old Mice; 18–20 month old	*Slc33a1, Snx17, Vps35, Cln3, Npc2, Grn*	LDAM are aged microglia with lipid droplets LDAM are defective in phagocytosis, LDAM produce reactive oxygen species and secrete pro-inflammatory cytokines.	[20]
Disease-associated microglia	DAM	Mice	Disease	5x FAD and mSOD G93A mice Approximately 6 months old	*Tyrobp, Ctsb, Ctsd, Apoe, B2m, Fth1, Lyz2, Trem2, Axl, Ctsl, Lpl, Cd9, Csf1, Ccl6, Itgax, Clec7a, Lilrb4, Timp2*	DAM appear in the disease model mice (5x FAD and mSOD G93A) DAM appearance occurs sequentially in a Trem2-independent and Trem2-dependent manner. DAM activation is required for downregulation of checkpoint genes (Cx3cr1, P2ry12, P2ry13).	[21]
Microglial neurodegenerative phenotype	MGnD	Mice	Disease	APP-PS, mSOD1 G93A, and EAE mice 2, 3, 4, 9, 20 and 24 months old	*Spp1, Itgax, Axl, Lilrb4, Clec7a,* *Csf1, Apoe*	MGnD are induced by neuritic Aβ plaque and apoptotic neurons. Trem2-ApoE pathway promotes a swith from homeostatic microglia to MGnD.	[22]
Human AD microglia	HAM	Human Mice	Disease	Human; 64 ± 16 years, 77 ± 17 years Mice; 4, 12 and 22 months	*Apoe, Abca7, Gpr141, Ptk2b,* *Spl1, Zyx*	HAM were identified from frozen cerebrocortical tissues from human AD brain. HAM profile is entirely distinct from the DAM profile defined in mouse models.	[23]
Microglia inflamed in MS	MIMS	Human	Disease	30–60 years	*Trem2, Apoe, Lpl, Cd68, Cd9, Cd74, Grn, Tyrobp, Timp2, Spp1, Ctsz, Ctsb, Fth1, C1qa, C1qb, C1qc*	MIMS appears in demyelinated white matter lesions. MIMS share a gene signature with other neurodegenerative diseases. C1q acts as a critical mediator of MIMS activation.	[24]
Human PD microglia	_	Human	Disease	>60 years old	*Il1b, Gpnmb, Hsp90aa1*	The nigral microglia in PD show a pro-inflammatory state.	[25]
Glioma-associated microglia	GAM	Human	Disease	Approximately 10 years old	*Itgal/Cd11a*	Itgal/CD11a is a novel marker for GAM. Itgal/CD11a ablation inhibits the growth of NF1 LGG.	[26]
Activated-response microglia Transiting response microglia Interferon response microglia Cycling/proliferating microglia	ARM TRM IRM CPM	Mice	Disease	APP NL-G-F mice APP/PS1-Apoe null mice 3, 6, 12, and 21 months old	*ARM; Cst7, Clec7a, Itgax, MHC class II, Cd74, H2-Ab1, H2-Aa, Ctsb, Ctsd, Spp1, Gpnmb, Dkk2* *TRM; similar to ARM* *IRM; Ifit2, Ifit3, Ifitm3, Irf7, Oasl2* *CPM; Top2a, Mcm2, Tubb5, Mki67, Cdk1*	ARM are enriched with AD risk genes. IRM show a high expression of genes involved in innate immune response. CPM enriched in genes involved in DNA replication, chromatin rearrangement, and cell cycle.	[40]

## Data Availability

Not applicable.

## Abbreviations

BAMs, Border-associated macrophages; CAMs, CNS-associated macrophages; EMPs, Erythromyeloid progenitors; DAM, disease-associated microglia; PAM, Proliferative-region-associated microglia; ATM, Axon tract-associated microglia; IRM, Injury-responsive microglia; WAM, White matter-associated microglia; LDAM, Lipid-droplet accumulating microglia; MGnD, Microglial neurodegenerative phenotype; HAM, Human AD microglia; MIMS, Microglia inflamed in MS; GAM, Glioma-associated microglia; ARM, Activated-response microglia; TRM, Transiting response microglia; IRM, Interferon response microglia; CPM, Cycling/proliferating microglia; AD, Alzheimer’s disease; PD, Parkinson’s disease; ALS, amyotrophic lateral sclerosis; MS, multiple sclerosis; CSF, cerebrospinal fluid; SLYM, subarachnoid lymphatic-like membrane; BCSFB, blood–CSF barrier; LPS, lipopolysaccharide.
